# Nomophobia: A Cross-Sectional Study of Lifestyle-Induced Factors Among Global Health Professionals

**DOI:** 10.1192/j.eurpsy.2024.1174

**Published:** 2024-08-27

**Authors:** F. A. Nawaz, A. Singh, P. Mehta, H. Matoung, S. Tawfeeq, D. Mahendru, A. Atulkar, D. Abraham, S. Padte, A. Kashyap, V. Monga, R. Shah, S. Surani, Z. Arshad, R. Kashyap

**Affiliations:** ^1^Global 3-P Research Group, Global Remote Research Scholars Program, MN, United States; ^2^Emirates Health Services, Al Amal Psychiatric Hospital, Dubai, United Arab Emirates; ^3^Department of Research, WellSpan Health, York, United States

## Abstract

**Introduction:**

In recent times, Health Professionals (HPs) people may feel a sense of discomfort and nervousness when disconnected from their smartphones, causing the emergence of the new phenomenon of “No Mobile Phone Phobia,” or Nomophobia.

**Objectives:**

We aim to study lifestyle-related factors that influence HPs’ Nomophobia.

**Methods:**

From April- June 2023, a global cross-sectional study was conducted using the modified Nomophobia questionnaire (NMP-Q). The original 20 NMP-Q questions (Qs) were reduced to 14 to avoid repetitive Qs with similar meanings. The Qs were categorized into 4 sections, A- Not Being Able to Access Information; B- Losing Connectedness; C- Not Being Able to Communicate; and D- Giving Up Convenience. A new section, “E- Daily Habits”, and “F- Smartphone Type”, and “Hours Spent Daily” were added. Before the launch, it was internally and externally validated by trained psychiatrists as well as experienced researchers. We utilized social media, WhatsApp, text and emails to share it with HPs of different specialties worldwide. The survey was anonymous and IRB-exempt.

**Results:**

Total 105 countries’ HPs participation led to 12,253 responses. Total 47.3% of HPs agreed/strongly agreed (A/SA) that they prefer to use their smartphone before bedtime. Over half (57.8%) of HPs A/SA checked their notifications immediately after waking up in the morning. Only 19.4 % of HPs A/SA that woke up in the middle of the night to check notifications. Total 40.5% of HPs A/SA, 22% were neutral, and 37.3% of HPs disagreed /strongly disagreed (D/SD) with using smartphones while eating their meals. A total of 52.7% of HPs preferred smartphone usage over exercising as a break, while 454.9% of HPs A/SA that they chose smartphones over exploring other hobbies for relaxation. A total of 44.2% of respondents A/SA with smartphone usage in the restroom, 39.8% D/SD. 37.4% of participants D/SD with getting distracted by notifications and resisted the urge to answer any calls or texts while performing a focused task, whereas 39.6% A/SA and 23% were neutral. A total of 80% of respondents met the modified criteria for moderate-severe nomophobia.

**Conclusions:**

In a large-scale survey-based study on Nomophobia, additional Qs in NMP-Q may help recognize that nomophobia can be a result of daily lifestyle decisions rather than an isolated issue.

**Disclosure of Interest:**

None Declared

